# The microglial translocator protein (TSPO) in Alzheimer’s disease reflects a phagocytic phenotype

**DOI:** 10.1007/s00401-024-02822-x

**Published:** 2024-11-14

**Authors:** Emma F. Garland, Henrike Antony, Laura Kulagowska, Thomas Scott, Charlotte Rogien, Michel Bottlaender, James A. R. Nicoll, Delphine Boche

**Affiliations:** 1 Clinical Neurosciences, Clinical and Experimental Sciences, Faculty of Medicine, University of Southampton, Southampton General Hospital, Southampton, SO16 6YD UK; 2 Paris-Saclay University, CEA, CNRS, Service Hospitalier Frederic Joliot, Orsay, Inserm, BioMaps France; 3 UNIACT Neurospin, CEA, Gif-Sur-Yvette, France; 4 Department of Cellular Pathology, University Hospital Southampton NHS Trust, Southampton, UK

**Keywords:** Translocator protein, Alzheimer’s disease, Microglia, Human, Inflammation

## Abstract

Translocator protein (TSPO) is a mitochondrial protein expressed by microglia, ligands for which are used as a marker of neuroinflammation in PET studies of Alzheimer’s disease (AD). We previously showed increasing TSPO load in the cerebral cortex with AD progression, consistent with TSPO PET scan findings. Here, we aim to characterise the microglial phenotype associated with TSPO expression to aid interpretation of the signal generated by TSPO ligands in patients. Human post-mortem sections of temporal lobe (TL) and cerebellum (Cb) from cases classified by Braak group (0–II, III–IV, V–VI; each *n* = 10) were fluorescently double labelled for TSPO and microglial markers: Iba1, HLA-DR, CD68, MSR-A and CD64. Quantification was performed on scanned images using QuPath software to assess the microglial phenotype of TSPO. Qualitative analysis was also performed for TSPO with GFAP (astrocytes), CD31 (endothelial cells) and CD163 (perivascular macrophages) to characterise the cellular profile of TSPO. The percentage of CD68^+^TSPO^+^ double-labelled cells was significantly higher than for other microglial markers in both brain regions and in all Braak stages, followed by MSR-A^+^TSPO^+^ microglia. Iba1^+^TSPO^+^ cells were more numerous in the cerebellum than the temporal lobe, while CD64^+^TSPO^+^ cells were more numerous in the temporal lobe. No differences were observed for the other microglial markers. TSPO expression was also detected in endothelial cells, but not detected in astrocytes nor in perivascular macrophages. Our data suggest that TSPO is mainly related to a phagocytic profile of microglia (CD68^+^) in human AD, potentially highlighting the ongoing neurodegeneration.

**Supplementary Information:**

The online version contains supplementary material available at 10.1007/s00401-024-02822-x.

## Introduction

The use of translocator protein (TSPO) radioligands and positron emission tomography (PET) scans are the current method to image neuroinflammation in living patients with neurological conditions such as Alzheimer’s disease (AD). The TSPO ligand binds primarily to microglia but binding to other central nervous system (CNS) cell types has also been reported [14]. Microglia are dynamic and plastic immune cells that adopt functions depending on the brain microenvironment [2]. In AD, microglia exhibit several patterns of reactivity based on the stage of disease [2, 12, 16], with a possible early protective vs. late inflammatory profile highlighted from TSPO PET studies [9, 15]. In preclinical models of AD, the microglial profile in the early stages have a homeostatic signature, identified with expression of genes such as purinergic receptor (*P2ry12*), C-X3-C motif chemokine receptor 1 (*Cx3cr1*) and transmembrane protein 119 (*Tmem119*) [20], and ramified morphology as observed with ionised calcium-binding adaptor 1 (Iba1) [3]. This homeostatic phenotype also appears to be present in brain areas that are not as affected by the disease, such as the cerebellum [12]. Whereas in late AD, microglial cells defined as disease-associated microglia (DAM) [20] or microglia associated with neurodegenerative disease (MGnD) [21] have upregulated genes such as triggering receptor expressed on myeloid cells 2 (*Trem2*) and apolipoprotein E (*ApoE*). In late-stage human AD, microglia have increased phagocytic/scavenging capability, with increased human leukocyte antigen-DR (HLA-DR), macrophage scavenging receptor-A (MSR-A) and cluster of differentiation 68 (CD68) expression, that are negatively associated with cognitive decline [24], and cells typically exhibit a less ramified / amoeboid morphology [3].

We, and others, have previously shown that TSPO expression increases as the disease progression worsens ex vivo, and this may be linked to the increase in phosphorylated (p)tau [12]. In vivo TSPO radioligand binding is increased in AD patients compared to controls [15, 16], and this increased uptake is associated with worsening cognitive decline [28]. However, a high level of TSPO expression seems to be indicative of a possible early protective phenotype, reflected by a high initial binding leading to a slower cognitive decline and vice versa [15, 16]. Application of TSPO ligands have demonstrated improved cognition in animal models also [5]. While it is known that TSPO radioligands bind primarily to microglia, it is unclear whether this marker identifies all microglia or a subset of microglia with a specific phenotype. TSPO is also expressed by other cell types such as endothelial cells and possibly astrocytes [12, 14, 16], which are involved in the pathogenesis of AD. As a result, this could confound the TSPO signal seen due to lower cell specificity. Furthermore, microglia can rapidly change their profile in disease [2] and show heterogeneity in different regions throughout the brain; therefore, TSPO may be associated with different subsets of microglia depending on disease stage and brain area.

This study aims to characterise the microglial immunoprofile associated with TSPO throughout the course of AD to better aid interpretation of PET scan results and to provide further understanding of which subset of microglia expresses TSPO.

## Materials and methods

### Cases

Human brain tissue from 30 donors was sourced from the South-West Dementia Brain Bank and matched as closely as possible for age, sex and post-mortem delay between the groups (Table 1). Cases were selected based on the Braak stage in order to allow exploration of the progression of the microglial profile and TSPO expression. Tissue from the middle/superior temporal gyrus and cerebellum was obtained for immunofluorescent analysis.

**Table 1:** Characteristics of the cases

Cases	Braak stage 0–II	Braak stage III–V	Braak stage V–VI
Sex	*4M:6F*	*5M:5F*	*5M:5F*
Age at death (years, mean ± SD)	*84.5* ± *9.5*	*89* ± *5.3*	*82* ± *7.9*
Braak stage	*0* = *3* *I* = *4* *II* = *3*	*III* = *5* *IV* = *5*	*V* = *5* *VI* = *5*
APOE genotype	*2/2* = *0*	*2/2* = *0*	*2/2* = *0*
	*2/3* = *2*	*2/3* = *2*	*2/3* = *0*
	*2/4* = *0*	*2/4* = *0*	*2/4* = *1*
	*3/3* = *6*	*3/3* = *5*	*3/3* = *3*
	*3/4* = *2*	*3/4* = *3*	*3/4* = *4*
	*4/4* = *0*	*4/4* = *0*	*4/4* = *1*
Post-mortem delay (hours, mean ± SD)	*47.3* ± *17.8*	*47.1* ± *20.1*	*33.9* ± *25.3*
Total	10	10	10

*M* male, *F* female, *SD* standard deviation

### Immunofluorescence

6 μm sections of formalin-fixed paraffin-embedded tissue were used to perform immunofluorescent double labelling to target TSPO (rabbit monoclonal, Abcam 109497) with ionised calcium-binding adaptor 1 (Iba1) (goat polyclonal, Abcam 5076), human leukocyte antigen (HLA)-DR (clone CR3/43, Dako M0775), CD68 (mouse monoclonal, Dako M0876), macrophage scavenger receptor (MSR)-A (goat polyclonal, R&D AF2708), CD64 (goat polyclonal, R&D AF1257), CD31 (mouse monoclonal, Abcam 9498), CD163 (mouse monoclonal, Bio-Rad MCA1853) and glial fibrillary acidic protein (GFAP) (mouse monoclonal, Abcam 4648) (Table 2). Antibodies were visualised using the appropriate fluorescently tagged secondary antibodies (ThermoFisher Highly Cross-Adsorbed Secondary Antibody, Alexa Fluor Plus 488/568/594). The sections were counterstained with DAPI (Sigma-Aldrich) and mounted with Mowiol (made in house). Negative controls with no primary antibody were included in all runs.

**Table 2:** Characteristics of the antibodies

Antibody	Species	Dilution	Supplier	Associated function/detection
TSPO	Rabbit	1:2500	Abcam (109497)	Microglial mitochondrial receptor
Iba1	Goat	1:1000	Abcam (5076)	Microglial motility and homeostasis
HLA-DR	Mouse	1:50	Dako (M0775)	Antigen presentation
CD68	Mouse	1:250	Dako (M0876)	Microglial phagocytosis
MSR-A	Goat	1:100	R&D (AF2708)	Microglial scavenging receptor with high affinity for Aβ
CD64	Goat	1:100	R&D (AF1257)	FCɣRI expressed by microglia
CD31	Mouse	1:50	Abcam (9498)	Endothelial cells
CD163	Mouse	1:500	Bio-Rad (MCA1853)	Perivascular macrophages
GFAP	Mouse	1:1000	Abcam (4648)	Reactive astrocytes

### Image acquisition and analysis

Scanned images of the staining were obtained with the Olympus VS110 automated slide scanner (Olympus America Inc.) at 20X magnification using DAPI, FITC and CY3 filter channels. Quantification of cell number (%) was based on Courtney and colleagues’ protocol [7]. For each slide, a region of interest (ROI) in a predetermined anatomical region of grey matter was selected in the QuPath software [1]. For the temporal lobe, all layers of grey matter were imaged, and for the cerebellum, images were obtained from both the granular and molecular layers. Positive cell detection was performed based on thresholds for each marker (Supplementary material). The positive cell detection tool was used to ascertain the percentage of cells for each marker individually (based on nuclei staining) or double stained (based on the microglial marker). TSPO labelled endothelial cells were manually discounted from the positive cell count. Each cell count was normalised to either total cell count or the corresponding microglial marker count and expressed as a percentage of co-occurrence for TSPO and each microglial marker within the cell. All analyses were performed in a blinded manner to case information.

### Statistical analysis

Statistical analysis was carried out using the IBM SPSS v28 statistical software package (SPSS Inc. Chicago IL) and GraphPad Prism v9.2 (GraphPad Software. San Diego CA) for the graphs. For each marker, normality of the distribution was assessed by the Shapiro–Wilk test, and the distribution was observed to be non-parametric for all markers, except MSR-A^+^TSPO^+^ in the temporal lobe and CD68^+^TSPO^+^ in the cerebellum, which were parametric. Comparisons between the different Braak stage groups and between the markers were carried out using the non-parametric Kruskal–Wallis test followed by Dunn’s post-hoc test if significant, or the parametric one-way ANOVA test with the Tukey’s post-hoc if significant. Comparisons between the temporal lobe and cerebellum were carried out using the non-parametric Mann–Whitney *U* test for all markers. *P* values less than 0.05 for intergroup comparisons were considered significant.

## Results

### Microglial cell count

**Fig. 1:**
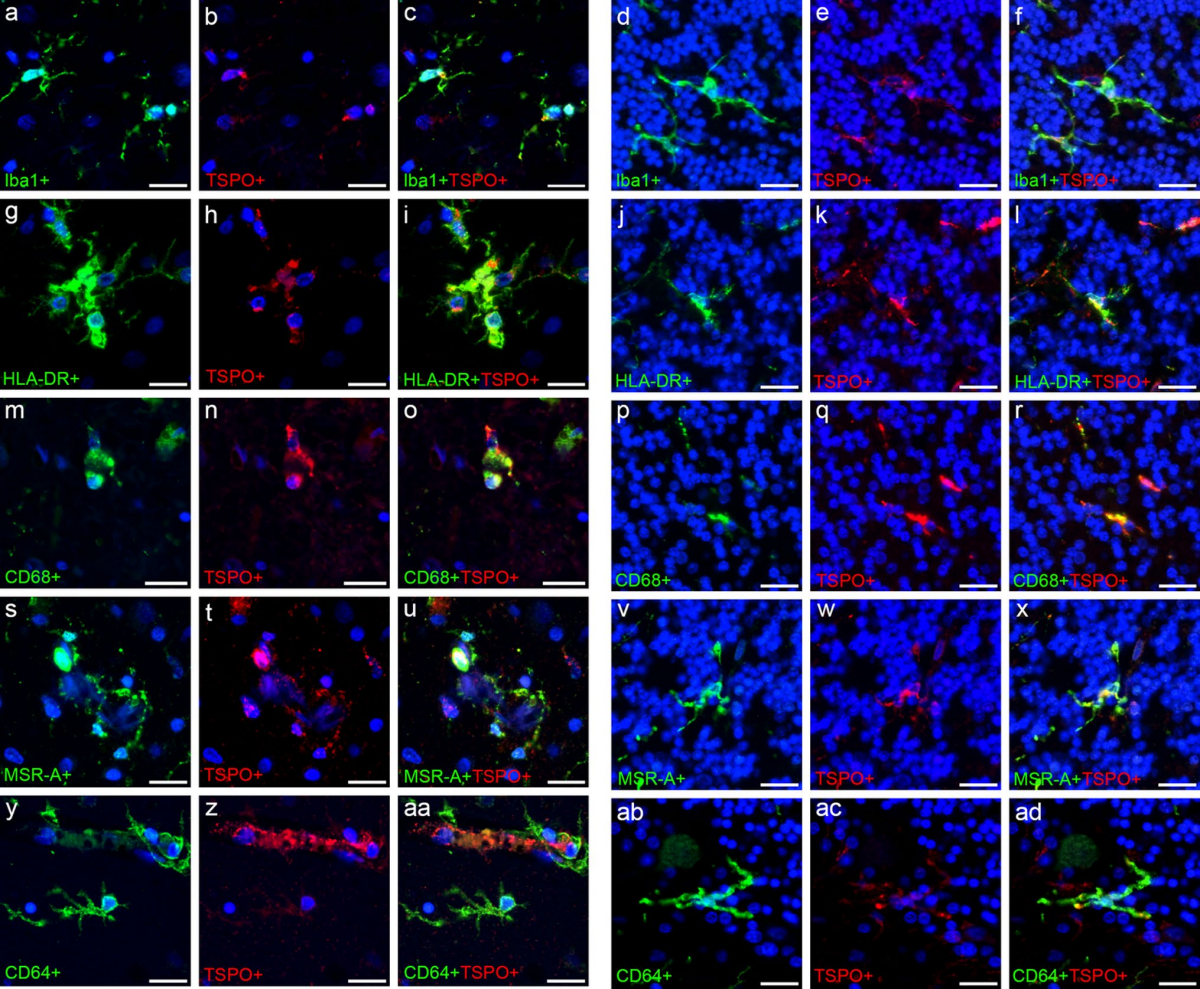
Detailed images of fluorescent double staining with microglial markers and TSPO. Single cell or cluster of cells positive for Iba1 (**a**–**f**), HLA-DR (**g**–**l**), CD68 (**m**–**r**), MSR-A (**s**–**x**) and CD64 (**y**–**ad**) (green) with TSPO^+^ cells (red) in the temporal lobe (**a**–**c**, **g**–**i**, **m**–**o**, **s**–**u**, **y**–**aa**) and cerebellum (**d**–**f**, **j**–**l**, **p**–**r**, **v**–**x**, **ab**–**ad**). Counterstained nuclei with DAPI (blue). Images are from Braak stage VI cases. Scale bars = 20 µm

TSPO co-occurrence with the microglial markers is primarily within the cell soma, with small amounts in the processes as observed with the microglial membrane markers (Iba1, HLA-DR, MSR-A and CD64) (Fig. 1). For CD68^+^TSPO^+^ staining, co-occurrence can be seen as both markers have a punctate pattern (Fig. 1m–r).

**Fig. 2:**
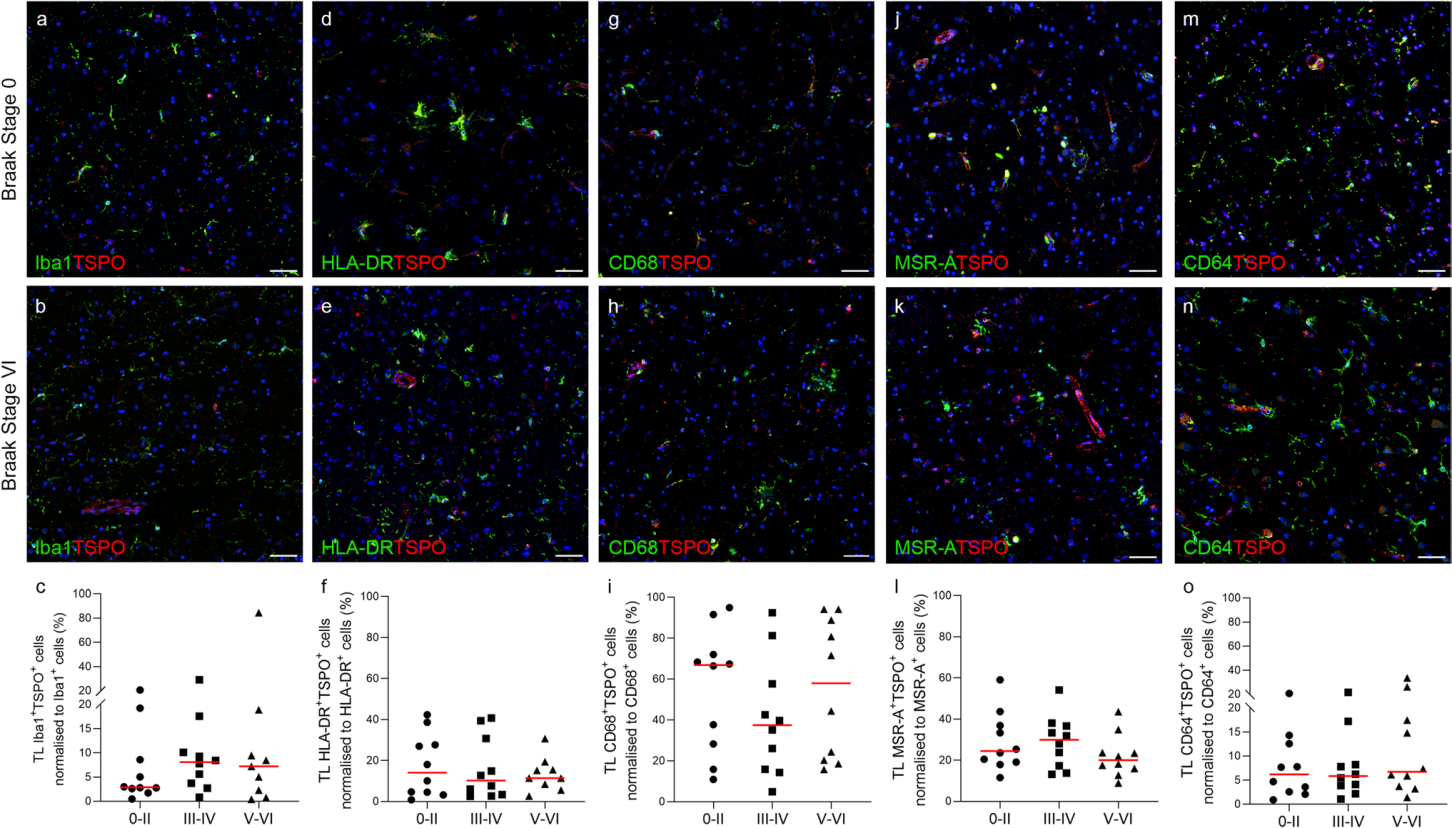
Fluorescent double labelling of microglial markers and TSPO in the temporal lobe. Images and quantification of Iba1^+^ (**a**–**c**), HLA-DR^+^(**d**–**f**), CD68^+^ (**g**–**i**), MSR-A^+^ (**j**–**l**) and CD64^+^ (**m**–**o**) microglial cells (green) with TSPO^+^ cells (red) normalised to corresponding microglial marker (%), presented by Braak group (0–II, III–IV, V–VI). Counterstained nuclei with DAPI (blue). Scale bars = 50 µm

**Fig. 3:**
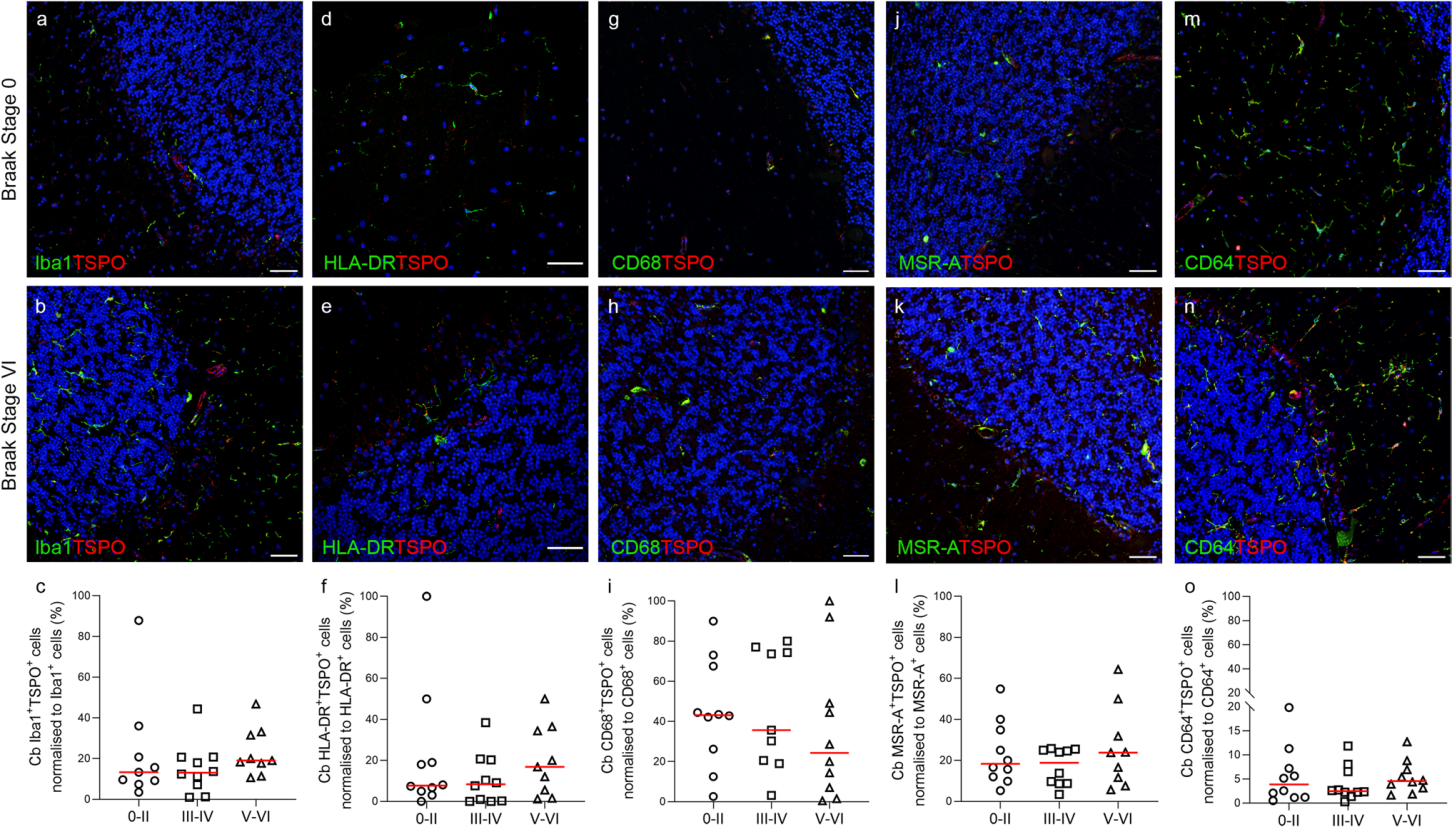
Fluorescent double labelling of microglial markers and TSPO in the cerebellum. Images and quantification of Iba1^+^ (**a**–**c**), HLA-DR^+^(**d**–**f**), CD68^+^ (**g**–**i**), MSR-A^+^ (**j**–**l**) and CD64^+^ (**m**–**o**) microglial cells (green) with TSPO^+^ cells (red) normalised to corresponding microglial marker (%), presented by Braak group (0–II, III–IV, V–VI). Counterstained nuclei with DAPI (blue). Scale bars = 50 µm

Quantification of the percentage of TSPO^+^ cells which co-express Iba1 (Iba1^+^TSPO^+^) showed no change in the temporal lobe (*P* = 0.5912) or cerebellum (P = 0.3123) over the course of the disease (Fig. 2c and 3c). This was also the case for: HLA-DR^+^TSPO^+^ cells (temporal lobe *P* = 0.9782; cerebellum *P* = 0.5178) (Figs. 2f and 3f), CD68^+^TSPO^+^ cells (temporal lobe *P* = 0.5171; cerebellum *P* = 0.7394) (Figs. 2i and 3i), MSR-A^+^TSPO^+^ cells (temporal lobe *P* = 0.3713; cerebellum *P* = 0.7106) (Fig. 2l and 3l), and CD64^+^TSPO^+^ cells (temporal lobe *P* = 0.6781; cerebellum *P* = 0.5976) (Figs. 2o and 3o). There were no changes in the percentage of single (Supplementary Fig. 1) or double cell counts (Supplementary Fig. 2) normalised to total cells across the Braak stages for any of the microglial markers, except for Iba1^+^TSPO^+^ cells in the cerebellum (*P* = 0.0377) (Supplementary Fig. 2). To confirm that our data were not affected by the overall number of cells changing over the course of disease, we performed analysis of the nuclei count across the Braak stages and found no significant difference (Supplementary Fig. 3).

### Microglial immunophenotype of cells labelled with TSPO

**Fig. 4:**
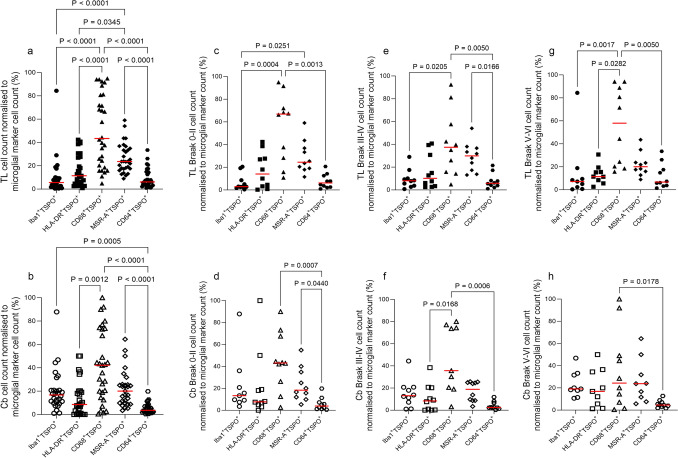
Comparisons of microglia double labelled with TSPO and different microglial markers by Braak stage. Cell counts normalised to microglial marker cell count (%), including the whole post-mortem cohort in the temporal lobe (**a**) and cerebellum (**b**) and then separated by Braak stage in the temporal lobe (**c**, **e**, **g**) and cerebellum (**d**, **f**, **h**)

To understand which marker, if any, had increased association with TSPO, comparisons were performed between the co-occurrence of TSPO and the different microglial markers, firstly independently of the Braak stage. The proportion of CD68^+^TSPO^+^ cells were the highest represented double-stained cells in both brain regions (temporal lobe: median 43.44%; cerebellum: median 42.15%), with their percentage significantly higher than Iba1^+^TSPO^+^ (*P* < 0.0001), HLA-DR^+^TSPO^+^ (*P* < 0.0001) and CD64^+^TSPO^+^ (*P* < 0.0001) cells in the temporal lobe, and higher than HLA-DR^+^TSPO^+^ (*P* = 0.0012) and CD64^+^TSPO^+^ (*P* < 0.0001) cells in the cerebellum (Fig. 4a and b). The second highest double-stained cell population was MSR-A^+^TSPO^+^ cells in both regions (temporal lobe: median 23.62%; cerebellum: median 20.00%) which was significantly higher than Iba1^+^TSPO^+^ (*P* < 0.0001), HLA-DR^+^TSPO^+^ (*P* = 0.0345) and CD64^+^TSPO^+^ (*P* < 0.0001) cells in the temporal lobe, and CD64^+^TSPO^+^ (*P* < 0.0001) cells in the cerebellum (Fig. 4a and b). The proportion of Iba1^+^TSPO^+^ cells was significantly higher than CD64^+^TSPO^+^ cells (*P* < 0.0001) in the cerebellum but not in the temporal lobe (Fig. 4b). Iba1^+^TSPO^+^ cells were the lowest population in the temporal lobe (median 5.59%) and CD64^+^TSPO^+^ cells the lowest in the cerebellum (median 3.53%).

When the cases were split by Braak stage group, a similar pattern of difference was seen.

In the temporal lobe: Braak stage 0–II exhibited the highest cell population for CD68^+^TSPO^+^ (median 66.9%), which was significantly higher than Iba1^+^TSPO^+^ (*P* = 0.0004) and CD64^+^TSPO^+^ (*P* = 0.0013) cells (Fig. 4c). The next highest double-stained cell percentage was MSR-A^+^TSPO^+^ (median 24.47%), which was significantly higher than Iba1^+^TSPO^+^ cells (*P* = 0.0251) (Fig. 4c). The lowest cell population was Iba1^+^TSPO^+^ (median 2.89%) (Fig. 4c).

In Braak stage III–IV, CD68^+^TSPO^+^ was the highest represented cells (median 37.43%) and was significantly higher than Iba1^+^TSPO^+^ (*P* = 0.0205) and CD64^+^TSPO^+^ (*P* = 0.005) cells (Fig. 4e). Then, MSR-A^+^TSPO^+^ cells were the next most prevalent (median 29.91%) and were significantly more than CD64^+^TSPO^+^ cells (*P* = 0.0166) (Fig. 4e). The lowest percentage of double-stained cells in this Braak group were CD64^+^TSPO^+^ cells (median 5.84%) (Fig. 4e). In Braak stage V–VI, the highest cell population remained CD68^+^TSPO^+^ (median 57.99%), which was significantly higher than Iba1^+^TSPO^+^ (*P* = 0.0017), HLA-DR^+^TSPO^+^ (*P* = 0.0282) and CD64^+^TSPO^+^ (*P* = 0.005) cells (Fig. 4g).

In the cerebellum: Braak stage 0–II displayed CD68^+^TSPO^+^ with the highest cell percentage (median 43.17%) and was significantly higher than CD64^+^TSPO^+^ (*P* = 0.0007) (Fig. 4d). MSR-A^+^TSPO^+^ was the next highest cell population (median 18.33%), which was significantly higher than CD64^+^TSPO^+^ cells (*P* = 0.044) (Fig. 4d) and was the lowest population in this group (median 3.86%) (Fig. 4d).

Braak stage III–IV exhibited a similar pattern with CD68^+^TSPO^+^ cells being the highest (median 35.71%), significantly higher than HLA-DR^+^TSPO^+^ (*P* = 0.0168) and CD64^+^TSPO^+^ (*P* = 0.0006) cells (Fig. 4f).

In Braak stage V–VI, there was a significant difference between CD68^+^TSPO^+^ and CD64^+^TSPO^+^ (*P* = 0.0178) cells with CD68^+^TSPO^+^ being the most prevalent (median 24.29%) and CD64^+^TSPO^+^ the lowest cell population (median 4.57%) (Fig. 4h).

**Fig. 5:**
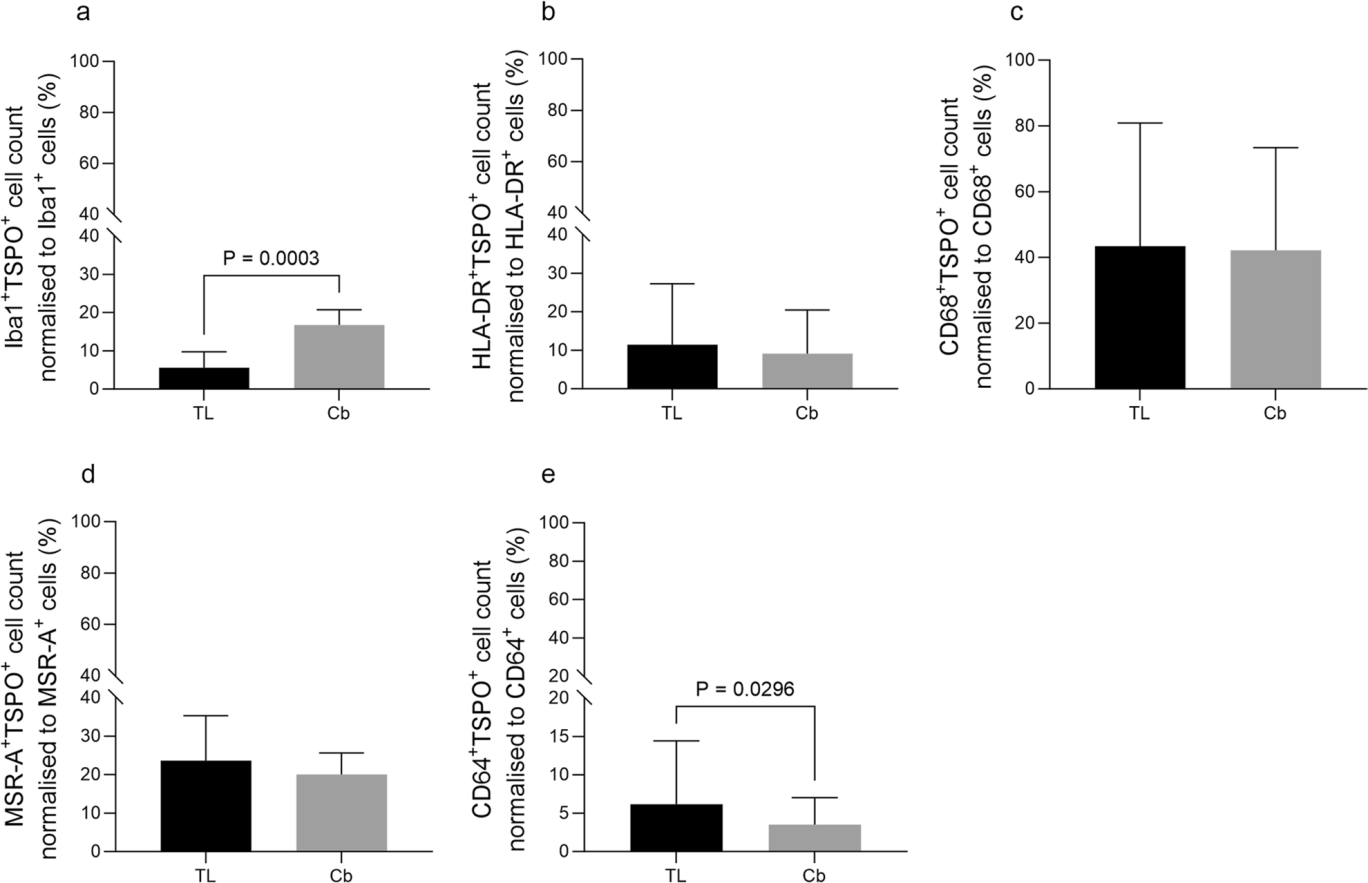
Comparisons between temporal lobe (TL) and cerebellum (Cb). Double labelling cell counts normalised to microglial marker cell count, including the whole post-mortem cohort

Interestingly, when comparing the percentage of cells in the temporal and cerebellum for the double labelling of each set of staining, there was no significant difference for HLA-DR^+^TSPO^+^, CD68^+^TSPO^+^ or MSR-A^+^TSPO^+^ cell percentages (Fig. 5b, c, d). This provides further evidence that while CD68^+^TSPO^+^ was the highest compared to other markers, this was independent of brain region. There were significantly more Iba1^+^TSPO^+^ cells in the cerebellum than the temporal lobe (*P* = 0.0003) (Fig. 5a), and a significantly higher percentage of CD64^+^TSPO^+^ cells in the temporal lobe compared to the cerebellum. (Fig. 5e).

### TSPO expression in other cell types

**Fig. 6:**
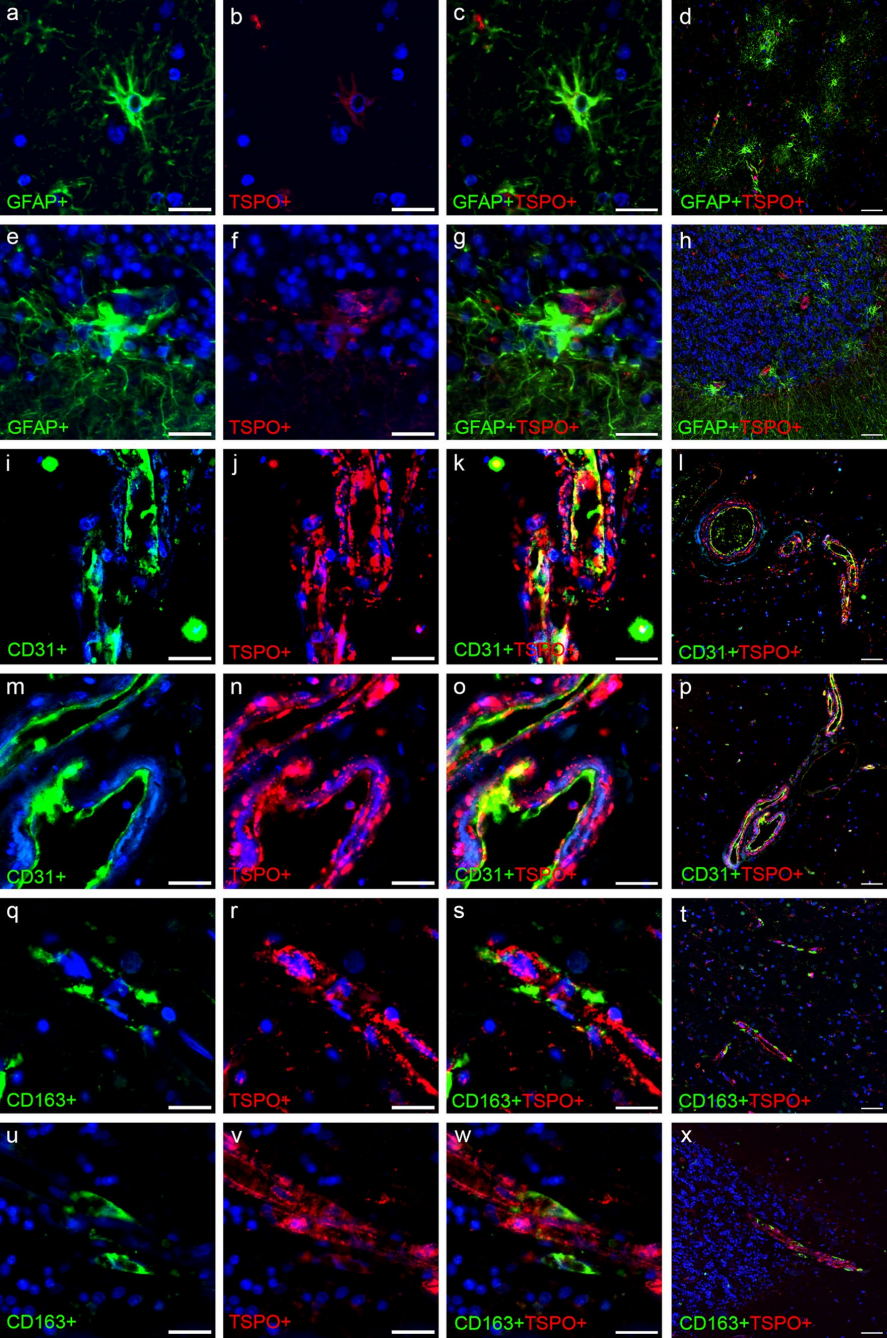
Fluorescent double staining of non-microglial markers and TSPO. GFAP^+^ astrocytes (green) (**a**–**h**), CD31^+^ endothelial cells (green) (**i**–**p**) and CD163^+^ perivascular macrophages (green) (**q**–**x**) with TSPO (red) in the temporal lobe (**a**–**d**, **i**–**l**, **q**–**t**) and cerebellum (**e**–**h**, **m**–**p**, **u**–**x**). Counterstained nuclei with DAPI (blue). Scale bars = 20 µm or 50 µm

Qualitative analysis of the non-microglial markers showed very little, if any, expression of TSPO in GFAP^+^ astrocytes or CD163^+^ perivascular macrophages (Fig. 6a–h and q–x). Co-occurrence of CD31^+^ endothelial cells with TSPO was detected (Fig. 6i–p). GFAP^+^ astrocytes were star shaped and abundant in the grey matter of the temporal lobe (Fig. 6d), while in the cerebellum, they appeared mainly clustered around the junction between granular and molecular layers of the grey matter (Fig. 6h). The TSPO staining in conjunction with GFAP was minimal within the astrocytes (Fig. 6a–h). The endothelial staining was predominantly located surrounding the lumen of the blood vessel with some co-occurrence of TSPO (Fig. 6i–p). Of note, TSPO expression was present throughout the whole of the blood vessel wall and not just localised to the intraluminal area (Fig. 6i–p), likely associated with smooth muscle cells. There was no co-occurrence of TSPO with CD163^+^ perivascular macrophages (Fig. 6q–x).

## Discussion

Within the human brain, microglia are highly dynamic and exhibit different morphologies and phenotypes, with some more prominent in disease [11, 20, 24]. In keeping with the finding that in vivo TSPO radioligand binding is increased in AD patients compared to controls [15, 16], we have previously shown histologically that TSPO expression increases with the progression of AD, and this may be linked to the increase in phosphorylated (p)Tau [12]. As TSPO PET scans are a tool used by clinicians to assess neuroinflammation in several neurological conditions, it is imperative to better understand the type of neuroinflammation associated with TSPO, and thus which microglial population TSPO highlights. With the use of microglial markers that are associated with known functions, we sought to elucidate the immunophenotype of TSPO-expressing microglia. We first show, using a semi-automated cell counting method, that the percentage of single and double-labelled microglia does not change over the progression of AD. This suggests that while microglia are able to change their morphology and immunophenotype, and in particular show an increased TSPO expression [12], the overall percentage of microglial cells and the percentage of TSPO^+^ microglia remains constant throughout the course of the disease, as reported by others [10, 27]. This may indicate a phenotypic change in microglia as a response to disease, rather than an increased cell number. The notion of a proliferative *vs* phenotypic change in AD is controversial, with some evidence in animal models suggesting that the former occurs [13, 19]. Other evidence suggests that there is solely a phenotypic change towards a reactive cell function and no change in microglial number [10, 29]. Finally, there is literature to suggest that both may occur in AD [17]. The whole picture is yet to be established but our data indicate that at least a disease-associated phenotypic alteration in microglial cells occurs.

We report that CD68^+^TSPO^+^ microglia were the more numerous TSPO^+^ microglial population in both the temporal and cerebellar regions. CD68 is expressed in the lysosomal compartment and is upregulated in phagocytic microglia [32]. CD68 expression has been associated with dementia and with poorer cognition in subjects with AD and in non-demented subjects [24]. This indicates that CD68 is linked with microglial reactivity, lending evidence to the notion that TSPO highlights reactive microglia rather than homeostatic/physiological cells. An interesting aspect of this finding is that the higher proportion of TSPO^+^ cells which expressed CD68 appeared to be independent of disease stage and brain region. This implies that TSPO expression is mainly related to phagocytosis and thus, as a maker for neuroinflammation, may also be associated with neurodegeneration in any region of the brain and/or at any stage of the disease. This provides clarification for the clinical interpretation of the microglial phenotype seen in TSPO PET scans. In AD, studies have reported increased TSPO binding in AD patients [9, 15, 16], which in conjunction with our findings implies increased reactive phagocytic/scavenging microglial cells, as corroborated by studies in human AD post-mortem tissue showing increased microglial CD68 expression [18, 24]. We previously showed, using an expanded version of the same cohort, that TSPO expression was associated with pTau in the temporal lobe [12]. Relating to this, CD68^+^ microglia have also been shown to be associated with the burden of tau, both in the form of tangles and neuritic plaques [24, 30]. Recently, a study challenged the use of TSPO as a marker of microglial activation in the human brain, although they did identify a weak association between TSPO and CD68 using analysis of publicly available human datasets [27], consistent with our findings. Overall, this evidence implies a close relationship between TSPO, CD68-related microglial phagocytosis, tau-related neurodegeneration and dementia [3].

The second highest association of TSPO^+^ microglia was with MSR-A. MSR-A is a scavenging receptor with a high affinity for Aβ [24], promoting the idea of TSPO being associated with a reactive scavenging microglial phenotype. Furthermore, the percentage of cells showing co-occurrence of TSPO and Iba1 were higher in the cerebellum than the temporal lobe, corroborating our previous work showing a more homeostatic microglial environment in this region [12], used as a pseudo-reference area for TSPO PET scans [23].

Finally, it is important to examine other cell types that may potentially express TSPO as this will influence the clinical interpretation of TSPO PET scans. We confirm TSPO expression in endothelial cells [14, 26]. Of note, endothelial cells and microglia share a non-neuroectodermal developmental origin [8] which could explain the expression of TSPO in both cell types, but being largely absent from other cells in the brain. However, the number of microglia in the brain is significantly higher than endothelial cells [25]; therefore, TSPO binding seen in PET analysis would predominantly represent microglial cells. Astrocytic expression of TSPO remains controversial with some studies confirming expression in astrocytes [14, 31], and others did not [6, 22]. Our finding supports the latter, with very little, if any, TSPO present in astrocytes. Finally, TSPO was not expressed by the perivascular macrophages. While this finding might appear unexpected, the absence of TSPO in the macrophages is in accordance with microglia and macrophages being two distinct populations with specific surface protein expression patterns [4].

## Conclusion

We sought to characterise the expression of TSPO in relation to microglial markers, in order to provide clarification of the phenotype associated with TSPO in this cell type. Here, we report no change in the percentage of microglia expressing TSPO over the course of AD in the temporal lobe and cerebellum. However, TSPO appears to be associated with a phagocytic/scavenging microglial phenotype, which is independent of disease stage and brain region. We also confirm the potential involvement of the endothelial cells in the PET signal with minimal contribution from astrocytes and perivascular macrophages. Our study suggests that the signal seen via TSPO PET scans in AD patients identifies phagocytic microglia, which are known to be associated particularly with tau pathology and the ongoing neurodegeneration.

## Supplementary Information

Below is the link to the electronic supplementary material.

Supplementary file1 (PDF 478 KB)

## Data Availability

The data that support the findings of this study are available from the corresponding author, upon reasonable request.

## Abbreviations

AD: Alzheimer’s disease
CD: Cluster of differentiation
CNS: Central nervous system
DAM: Disease-associated microglia
GFAP: Glial fibrillary acidic protein
HLA-DR: Human leukocyte antigen—DR
Iba1: Ionised calcium-binding adaptor molecular 1
MGnD: Microglia associated with neurodegenerative disease
MSR-A: Macrophage scavenging receptor—A
PET: Positron emission tomography
pTau: Phosphorylated tau
ROI: Region of interest
TSPO: Translocator protein
