# Supplementary material for: The microglial translocator protein (TSPO) in Alzheimer’s disease reflects a phagocytic phenotype
Source: Acta Neuropathol. 2024 Nov 14;148(1):62. doi: 10.1007/s00401-024-02822-x (PMC11564344; doi:10.1007/s00401-024-02822-x)
Supplement: Supplementary file 1 — Supplementary file1 (PDF 478 KB) [file 401_2024_2822_MOESM1_ESM.pdf]

The microglial translocator protein (TSPO) in Alzheimer's disease reflects a phagocytic phenotype.

Emma F. Garland[1], Henrike Antony[1], Laura Kulagowska[1], Thomas Scott[1], Charlotte Rogien[1], Michel Bottlaender[2,3], James A.R. Nicoll[1,4], Delphine Boche[1].

[1]Clinical Neurosciences, Clinical and Experimental Sciences, Faculty of Medicine, University of Southampton, Southampton, UK

[2]Paris-Saclay University, CEA, CNRS, Inserm, BioMaps, Service Hospitalier Frederic Joliot, Orsay, France

[3]UNIACT Neurospin, CEA, Gif-sur-Yvette, France

[4]Department of Cellular Pathology, University Hospital Southampton NHS Trust, Southampton, UK

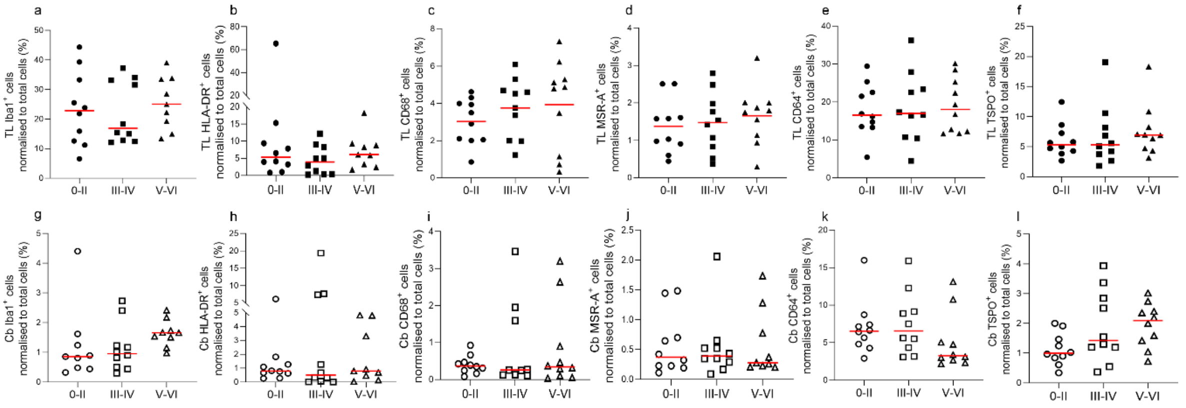

Supplementary Figure 1. Quantification of positive cell counts for Iba1 (**a, g**), HLA-DR (**b, h**), CD68 (**c, i**), MSR-A (**d, j**), CD64 (**e, k**) and TSPO (**f, l**) normalised to total cells (%) in the temporal lobe (**a-f**) and cerebellum (**g-l**), separated by Braak group (0-VI).

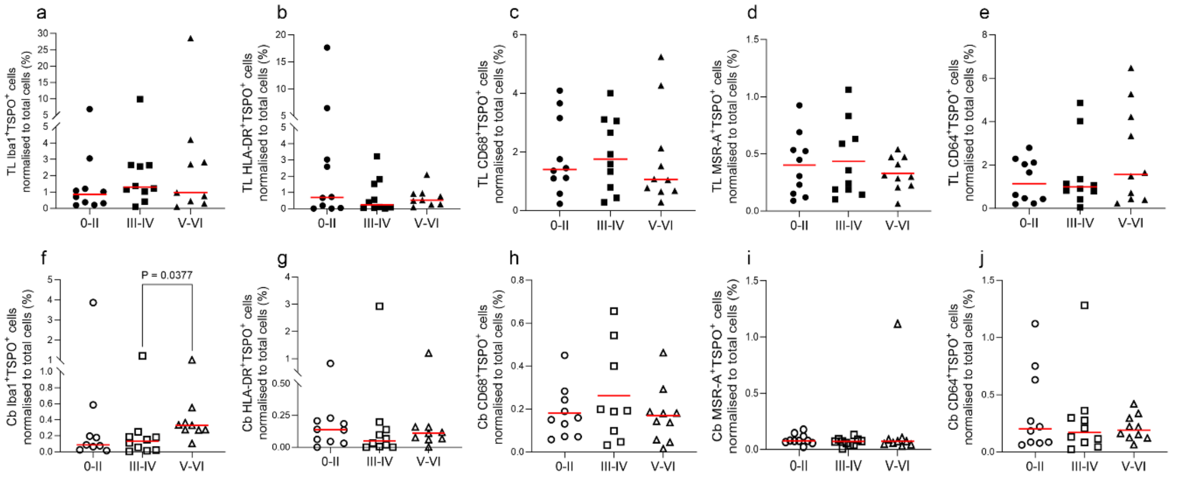

Supplementary Figure 2. Quantification of positive cell counts for Iba1 (**a, f**), HLA-DR (**b, g**), CD68 (**c, h**), MSR-A (**d, i**) and CD64 (**e, j**) with TSPO, normalised to total cells (%) in the temporal lobe (**a-e**) and cerebellum (**f-j**), separated by Braak group (0-VI).

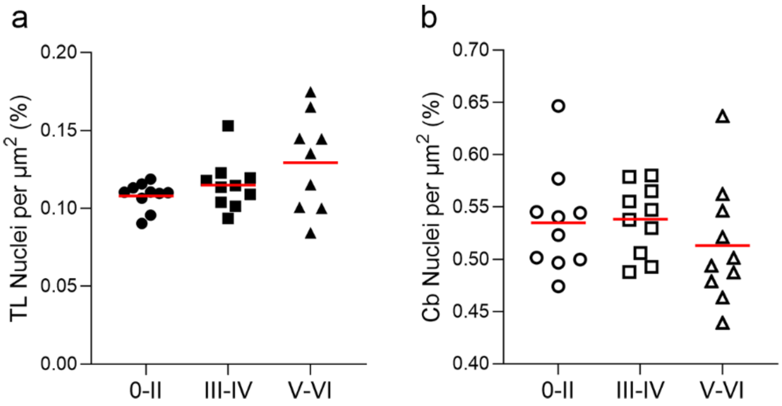

Supplementary Figure 3. Quantification of nuclei counted (DAPI) per μm^2 (%) in the temporal lobe (**a**) and cerebellum (**b**), separated by Braak group (0-VI).

Supplementary Table 1. QuPath thresholds for positive cell counts of Iba1 (FITC) and TSPO (CY3)

**Temporal**	DAPI+FITC	DAPI+CY3	FITC+CY3	**Cerebellum**	DAPI+FITC	DAPI+CY3	FITC+CY3
Background radius	3µm	3µm	3µm	Background radius	3µm	3µm	3µm
Min area	10µm²	10µm²	10µm²	Min area	10µm²	10µm²	10µm²
Max area	100µm²	100µm²	100µm²	Max area	100µm²	100µm²	32µm²
DAPI threshold	80	80	250	DAPI threshold	80	80	200
Cell expansion	1µm	2µm	3µm	Cell expansion	1µm	2µm	3µm
Threshold +1	250	300	300	Threshold +1	200	1000	1000

Supplementary Table 2. QuPath thresholds for positive cell counts of HLA-DR (FITC) and TSPO (CY3)

**Temporal**	DAPI+FITC	DAPI+CY3	FITC+CY3	**Cerebellum**	DAPI+FITC	DAPI+CY3	FITC+CY3
Background radius	3μm	3μm	5μm	Background radius	3μm	3μm	5μm?
Min area	10μm²	10μm²	10μm²	Min area	10μm²	10μm²	10μm²
Max area	100μm²	100μm²	100μm²	Max area	100μm²	100μm²	100μm²
DAPI threshold	120	120	250/400	DAPI threshold	100	100	400/300
Cell expansion	1μm	2μm	3μm	Cell expansion	1μm	2μm	3μm
Threshold +1	500/400	550/350	480/350	Threshold +1	600/400	550/350	480/350

Supplementary Table 3. QuPath thresholds for positive cell counts of CD68 (FITC) and TSPO (CY3)

**Temporal**	DAPI+FITC	DAPI+CY3	FITC+CY3	**Cerebellum**	DAPI+FITC	DAPI+CY3	FITC+CY3
Background radius	3µm	3µm	3µm	Background radius	3µm	3µm	3µm
Min area	10µm²	10µm²	10µm²	Min area	10µm²	10µm²	10µm²
Max area	100µm²	100µm²	100µm²	Max area	100µm²	100µm²	100µm²
DAPI threshold	120	120	400	DAPI threshold	120	120	400
Cell expansion	2µm	2µm	3µm	Cell expansion	2µm	2µm	3µm
Threshold +1	400	650	650	Threshold +1	400	650	650

Supplementary Table 4. QuPath thresholds for positive cell counts of MSR-A (FITC) and TSPO (CY3)

**Temporal**	DAPI+FITC	DAPI+CY3	FITC+CY3	**Cerebellum**	DAPI+FITC	DAPI+CY3	FITC+CY3
Background radius	3µm	3µm	3µm	Background radius	3µm	3µm	3µm
Min area	10µm²	10µm²	10µm²	Min area	10µm²	10µm²	10µm²
Max area	100µm²	100µm²	100µm²	Max area	100µm²	100µm²	100µm²
DAPI threshold	80	80	600	DAPI threshold	80	80	600
Cell expansion	1µm	2µm	3µm	Cell expansion	1µm	2µm	3µm
Threshold +1	600	450	450	Threshold +1	600	900	900

Supplementary Table 5. QuPath thresholds for positive cell counts of CD64 (FITC) and TSPO (CY3)

**Temporal**	DAPI+FITC	DAPI+CY3	FITC+CY3	**Cerebellum**	DAPI+FITC	DAPI+CY3	FITC+CY3
Background radius	3μm	3μm	3μm	Background radius	3μm	3μm	3μm
Min area	10μm²	10μm²	10μm²	Min area	10μm²	10μm²	10μm²
Max area	100μm²	100μm²	100μm²	Max area	100μm²	100μm²	100μm²
DAPI threshold	80	80	450	DAPI threshold	80	80	450
Cell expansion	1μm	2μm	3μm	Cell expansion	1μm	2μm	3μm
Threshold +1	450	500	500	Threshold +1	450	550	550
